# Structural Variants as a Basis for Targeted Therapies in Hematological Malignancies

**DOI:** 10.3389/fonc.2019.00839

**Published:** 2019-08-28

**Authors:** Judith Schütte, Julia Reusch, Cyrus Khandanpour, Christine Eisfeld

**Affiliations:** ^1^Department of Medicine A, Hematology, Oncology and Pneumology, University Hospital Münster, Münster, Germany; ^2^Medizinische Fakultät, Universität Münster, Münster, Germany

**Keywords:** structural variant (SV), hematology, targeted therapy, chromosomal rearrangements, chromatin structure

## Abstract

Structural variants (SV) are changes in the genomic landscape that can alter gene expression levels and thus lead to disease development. The most common and best studied SVs in hematological malignancies are chromosomal translocations. Here, parts of two genes that are normally on different chromosomes come into close proximity due to a failure in DNA repair. As a consequence, fusion proteins which show a different function and/or cellular localization compared to the two original proteins are expressed, sometimes even at different levels. The identification of chromosomal translocations is often used to identify the specific disease a patient is suffering from. In addition, SVs such as deletions, duplications, inversions and single nucleotide polymorphisms (SNPs) can occur in hematopoietic cells and lead to their malignant transformations. Changes in the 3D genome structure have also recently been shown to impact disease development. In this review, we describe a variety of SVs occurring in different subtypes of hematological malignancies. Currently, most therapeutic approaches target fusion proteins which are the cellular product of chromosomal translocations. However, amplifications and SNPs also play a role in disease progression and can be targeted. We present some examples for different types of structural variants and how they are currently treated.

## Introduction

Hematological malignancies are a group of diseases affecting the blood or immune system that are derived from either the myeloid or the lymphoid lineage, respectively. The most common myeloid malignancies are acute or chronic myeloid leukemia (AML or CML), myelodysplastic syndrome (MDS), and myeloproliferative neoplasms (MPN), while the most common lymphoid-derived diseases are acute or chronic lymphoid leukemia (ALL and CLL), lymphomas and multiple myelomas (1).

A major cause that is common to hematological malignancies is the presence of chromosomal rearrangements. The detection of copy number variations and other quantitative aberrations yielded insights into tumor pathogenesis in leukemias and lymphomas, and the recent development of high-throughput DNA sequencing allows the precise detection of chromosomal “breakpoints” (2–6). Chromosomal rearrangements typically translate into gene products with a deregulatory effect on proliferation and differentiation of tumor cells (7). More recently, growing knowledge about disease associated single nucleotide polymorphisms (SNPs) as well as variations affecting the 3D genome structure of malignant cells has extended the field of structural variation. The characterization of structural variants (SVs) and subsequent downstream mechanisms essentially improved the detection and classification of hematological malignancies and finally led to the development of targeted therapies.

## Chromosomal Translocations

Chromosomal translocations often oppose one gene to the regulatory region of another gene resulting in a fusion protein that gains aspects of both original proteins such as the DNA-binding domain of one and the protein-protein interaction domain of the other protein (8). As a consequence the fusion binding protein binds to DNA elements specific to the first protein and recruits proteins which are interaction partners of the second protein to these genomic regions. This in turn leads to deregulated gene expression of the target genes.

Chromosomal rearrangements frequently act as strong drivers of myeloid leukemogenesis. In childhood and adolescent AML, structural chromosomal aberrations such as fusions and translocations are often the only genomic variants detected, whereas in adult AML patients short variants and combinations of several independent mutations are more common (9). Translocations can also be found in other hematological diseases such as CML or Burkitt's lymphoma. In these entities they are powerful drivers of the disease. As they are present in the founding clone and are often genetically stable throughout the course of the disease, translocations and their transcripts serve as markers of the disease and allow for measurement of minimal residual disease (MRD), an indispensable tool to control for treatment response and to detect low level disease (10, 11).

**Figure 1:**
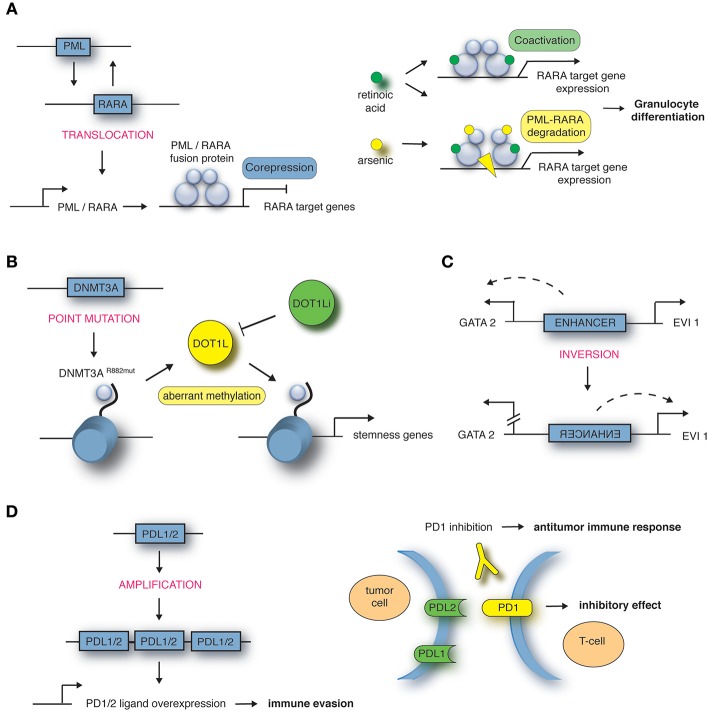
Mechanisms of structural variations in hematological malignancies. **(A)** Translocation t(15;17) encoding the PML-RARA fusion in APL, targeted by all-trans retinoic acid (ATRA) in combination with arsenic trioxide (ATO). Adapted from de Thé et al. (16). **(B)** Point mutation in DNMT3A gene locus in AML leads to aberrant methylation and recruitment of DOT1L, targeted by DOT1L inhibitor (DOT1Li). Adapted from Lu et al. (17) and Lu and Wang (18). **(C)** Inversion inv(3) encoding an enhancer in AML. Adapted from Bhagwat et al. (19). **(D)** Amplification of 9p24 in Hodgkin's lymphoma leads to PDL1/PDL2 overexpression, targeted by PD1 inhibitor. Adapted from Goodman et al. (20).

Exemplified, we are describing in the next paragraph some of the most common chromosomal rearrangements for the various hematological malignancies. It is important to note that this list is by no means complete. Well-known AML-causing translocations are the fusion of the transcription factors AML1 and ETO [t(8;21)], PML and RARα [t(15;17)] as well as MLL and AF9 [t(9;11)] (12). The t(8;21) translocation occurs in around 12% of AML cases and leads to a fusion of the transcription factor (TF) AML1 (also known as RUNX1) and the co-repressor Eleven-Twenty-One ETO (also known as MTG8) resulting in repression of RUNX1 target genes in a dominant-negative manner (8). A computational analysis of nine TFs in hematopoietic stem/progenitor cells demonstrates how deregulated expression of the fusion gene affects expression of the other eight TFs of this regulatory network, hence explaining in more depths the functional consequences (13). Around 98% of cases of acute promyelocytic leukemia (APL) are characterized by the juxtaposition of the promyelocytic leukemia gene (PML) and the retinoic acid receptor α (RARα) (14). RARα represses gene expression in the absence and activates gene expression in the presence of its ligand all-trans retinoic acid (ATRA). The PML-RARα fusion protein results in permanent repression of RARα target genes even in the presence of physiological levels of ATRA (15) (Figure 1A). The third example of AML-causing translocation are MLL (mixed lineage leukemia)-rearranged leukemias such as the fusion protein MLL-AF9 where the N-terminal part of the MLL gene is fused to AF9 (also known as MLLT3), a protein with a role in transcriptional activation. MLL-rearranged leukemias comprise around 5–10% of all AML cases and of these around 30% carry the t(9;11) translocation (21).

With regard to CML, blast cells harbor the t(9;22) translocation resulting in the fusion protein BCR-ABL1, known as the Philadelphia chromosome (22, 23). The fusion protein leads to a constitutively active tyrosine kinase. Burkitt's lymphoma, a non-Hodgkin lymphoma arising from B-cells, is associated with the t(8;14) translocation and is rapidly fatal if left untreated (24). Here, the gene locus of the transcriptional activator MYC is linked to the immunoglobulin heavy chain locus IGH. In multiple myeloma, one of the most common translocations also involves IGH. In this case the translocation t(11;14) leads to upregulation of Cyclin D1, thus affecting cell cycle regulation (25).

## Other Structural Variants

In recent years, other structural variations (SVs) than translocations have been discovered as an underlying or supporting mechanism for disease development and progression. These SVs include among others deletions, amplifications such as duplications, inversions, and single nucleotide polymorphisms (SNPs).

Deletions of the 11q23 region affecting the ATM gene have been discovered to play a role in a number of hematological malignancies such as B-cell chronic lymphocytic leukemia (B-CLL) and mantle cell lymphomas (MCL) [reviewed in (26, 27)]. Interestingly, not only protein-coding DNA sequences can be affected by deletions, but also regulatory genomic regions. In T-cell acute lymphoblastic leukemia (T-ALL), the deletion of a CTCF binding site destroys the three-dimensional (3D) genome structure around the TAL1 and LMO2 gene loci, resulting in gene activation through enhancers which are normally located in the neighboring topologically associated domains (TADs) (28). Another disease causing SV present in around 5% of T-ALL patients is a duplication of a NOTCH-bound enhancer region located on 8q24 regulating MYC expression (29).

Furthermore, next-generation sequencing highlighted that around 2% of the genome contains protein-coding genes. Single nucleotide polymorphisms (SNPs) are inherited substitutions of a single nucleotide which might affect the protein-coding sequence and hence its function. In some cases the presence of SNPs in genes regulating DNA repair, metabolism or cell cycle increases the probability of cancer development (30). For example, the transcriptional repressor GFI1 (growth factor independence 1) is in most cases comprised of the amino acid serine at position 36 (GFI1-36S), but in 3–7% of healthy whites a SNP leads to expression of the amino acid asparagine at this position (GFI1-36N). Humans carrying the GFI1-36N variant have an increased risk of developing AML (GFI1-36N present in 11% of AML patients) (31). Another gene which is mutated in around 20% of AML cases is DNA methyltransferase DNMT3A. A mutation at amino acid position 882 of DNMT3A results in a change from arginine to histidine leading to an altered methylation pattern at target genes (17), which results in the recruitment of DOT1L, another methyltransferase promoting transcriptional activation of “stemness” genes (Figure 1B). DOT1L inhibitors block the oncogenic program in DNMT3A mutated AML cells and have been introduced in early clinical trials (32, 33). Moreover, SNPs can also occur in upstream regulating regions creating novel TF binding sites causing disease development. A SNP located 4 kb upstream of the LMO1 transcriptional start site leads to a TCA to TTA (serine to leucine) exchange and hence creation of a MYB binding site. Consequently, LMO1 expression is deregulated through this novel oncogenic enhancer causing T-ALL development (34).

## 3D Genome Structure

To identify novel SVs in cancer patients, there have been a number of studies analyzing the 3D genome structure (35, 36). Hi-C and its derivates have been developed to detect DNA-DNA interactions in a genome-wide fashion and highlight the importance of the chromatin architecture in gene expression regulation. In a recent proof-of-principle study, the low-C method, a Hi-C method with low input material, was used to identify the common t(3;14) translocation in a patient with diffuse large B-cell lymphoma which affects BCL6 and IGH gene loci (35).

How alterations to the 3D genome structure influence disease development and progression was also shown by the identification of the inversion between 3q21 and 3q26 (37, 38). This SV leads to ectopic expression of the TF EVI1 which in turn leads to AML development. Upregulated EVI1 expression is mediated by a positional change of an enhancer which normally drives GATA2 expression, but is in close proximity to the EVI1 gene in inv(3) patients (Figure 1C). The relevance of enhancer hijacking by various SVs is highlighted by a number of studies describing this phenomenon in other cancer entities such as medulloblastoma (39) and salivary gland acinic cell carcinoma (AciCC) (40). For instance, the analysis of medulloblastoma genome sequencing data revealed that juxtapositioning of enhancers to neighboring genes leads to increased expression of oncogenes such as GFI1 or GFI1b in sub-groups of medulloblastoma cases (39).

## Detection of SV

The presence of SVs, especially translocations, can be evaluated by various techniques in order to identify the disease type as well as the best treatment option. PCR (polymerase chain reaction), FISH (fluorescence *in situ* hybridization) flow cytometry and SNP arrays are the best established techniques. PCR diagnostics is used to look for specific disease-causing genetic defects by amplifying specific target genes (41). It is relatively fast and cost-effective as a number of targets can be analyzed in parallel. In contrast, flow cytometry is used to analyse the surface marker expression of cells by detecting up to 10 parameters which can distinguish a cancer cell from a non-malignant cell (41, 42). Additionally, cell proliferation and cell cycle status which have been shown to be associated with disease progression can be determined by flow cytometry. Real-time PCR and flow cytometry can also be used to detect MRD, thus minor malignant cell populations (41–43). MRD diagnostics can hence be applied as a prognostic factor and allows for early detection of incipient relapse. In recent years, both techniques have been developed further to be more sensitive, to be applicable more broadly and to be more high-throughput (41, 42). Furthermore, SNP arrays are a form of DNA arrays in which you can identify single nucleotide exchanges as well as alterations from the diploidy of cells (44). Another method in routine diagnostics is FISH (43). Here, fluorescent probes are used to locate the DNA counterpart of the probe sequence in a biological sample. It thus allows the identification of specific DNA fragments on chromosomes. If two fluorescent probes that are usually on different chromosomes are detected in close proximity, it indicates a translocation event.

A novel approach to identify new SVs in cancers including 3D genome structure, deletions and amplifications is the development of chromosome conformation capture methods such as 3C, 4C, 5C, Hi-C, or Capture-C (45). Hi-C and its derivates were developed to detect DNA-DNA interactions in a genome-wide fashion and highlight the importance of the chromatin architecture in gene expression regulation. In a recent proof-of-principle study, the low-C method, a Hi-C method with low input material, was used to identify the common t(3;14) translocation in a patient with diffuse large B-cell lymphoma which affects BCL6 and IGH gene loci (35). Together with Hi-C data in other human tumor types (36), the study by Diaz et al. demonstrate that these genome-wide methods can represent a novel approach to identify new structural variants in cancers, but these approaches are currently not yet cost-effective.

## Examples for Targeted Therapies

### Targeting BCR-ABL1 in CML

In CML, the translocation t(9;22) (q34;q11) leads to the constitutively active fusion protein BCR-ABL1 tyrosine kinase which transfers phosphate from ATP to tyrosine residues of various substrates and finally leads to massively increased proliferation and decrease of apoptosis in myeloid precursor cells via multiple downstream signaling pathways (46). Importantly, the cytoplasmic location of the BCR-ABL1 oncoprotein allows interference with many cellular substrates which are inaccessible to the predominantly nuclear ABL protein (47). In a mouse model it could be demonstrated that BCR-ABL1 is the major pathogenic molecular event in CML (2). BCR-ABL1 is also exhibited in a subset of adult ALL, in some MPN and in rare cases of AML. The large majority of patients are diagnosed in the chronic phase (CP), characterized by excess numbers of immature cells at different stages of myelopoiesis which are capable to differentiate and preserve their functionality. In the natural course of disease, the enhanced proliferation is associated with genetic instability on the cytogenetic and on the nucleotide level, contributing to disease evolution to the blast phase (BP). The proportion of patients exhibiting typical additional chromosomal aberrations at diagnosis in CML-CP is 5%, but rises during the course of disease to 80% in CML-BP (48). The tyrosine kinase inhibitor (TKI) imatinib competitively blocks the ATP-binding site of the ABL kinase domain, thereby blocking downstream pathways. In addition, it also targets the PDGFR (platelet-derived growth factor receptor) and c-KIT kinases. The introduction of imatinib in newly diagnosed CML-CP patients could demonstrate overall survival and progression free survival rates at 10 years of 83 and 92%, respectively (49). While these results have been obtained under continuous therapy, an increasing body of evidence supports treatment discontinuation in patients who experienced long-lasting molecular remissions under TKI therapy, hence the goal of chemotherapy-free healing of CML is approaching (50–52). Second generation TKI, namely dasatinib, nilotinib, and bosutinib are more potent than imatinib at inhibiting BCR-ABL1 and in addition to being effective in patients resistant or intolerant to imatinib, they have shown to decrease the time to major molecular responses (MMR) compared to imatinib (53, 54). Although successful evolution of therapies leads to long-term remission in the majority of patients, a subset fails to achieve pre-determined levels of remission or exhibit increasing BCR-ABL1 transcripts after initial response, reflecting primary or secondary TKI resistance. Mechanisms of resistance most frequently involve point mutations of the ABL kinase domain, such as the highly resistant T315I mutation. As none of the second generation TKI is effective in T315I mutated CML, the development of the third generation TKI ponatinib which inhibits all documented BCR-ABL1 kinase domain mutants including T315I was an important milestone (55). Other mechanisms of resistance include BCR-ABL1 over-expression through the formation of extra copies of Philadelphia chromosomes and iso-derived Philadelphia chromosomes, as well as secondary genetic aberrations resulting in BCR-ABL1 independent proliferation (56, 57). While life expectancy in the majority of CML-CP patients is in a normal range due to TKI therapy, treatment of TKI-resistant CML and advanced-stage CML remains a major challenge.

### Targeting PML-RARα in APL

The translocation t(15;17) encoding the PML-RARα fusion is the prevailing genomic abnormality and most often the only driver mutation in APL. Therapeutic doses of ATRA can stop the PML-RARα-mediated repression of target genes and release the differentiation block in promyelocytes (58), sometimes catalyzing a dynamic process clinically apparent as APL differentiation syndrome. While ATRA-induced differentiation alone cannot cure APL, the addition of ATRA to anthracycline-based chemotherapy improved outcome (59, 60). Eventually, the replacement of chemotherapy by arsenic trioxide (ATO) in combination with ATRA in frontline therapy led to outstanding survival rates with remarkable reduction of chemotherapy-related toxicity (61). ATO targets the PML-moiety of PML-RARα, leading to proteolysis of the oncoprotein, which might also contribute to differentiation (62) (Figure 1A). Chemo-free therapy with ATRA-ATO is now regarded as standard treatment of non-high-risk APL (63). The question of whether high-risk APL patients with WBC count >10 × 10^9^/L equally benefit from a chemotherapy-free regimen has not been conclusively answered yet. Besides its role as a therapeutic target, PML-RARα serves as a biomarker specific to the disease. PCR-based techniques allow rapid diagnosis and measurement of residual disease (MRD) with distinct prognostic implications, as the absence of detectable transcripts is a precondition for long-term survival (64).

### Targeting PDL1 in Hodgkin's Lymphoma

Classic Hodgkin's lymphomas include small numbers of malignant Reed-Sternberg cells within an extensive but ineffective inflammatory and immune cell infiltrate (65). One way of these malignant cells to evade immune detection and deletion is to impede T-cell response. A key approach to silence T-cell response is upregulation of programmed cell death (PD) ligand 1 and 2 (20). The genes encoding the PD-1 ligands, PDL1 and PDL2, are located on chromosome 9p24.1. Amplification of these gene segments is a recurrent genetic abnormality in the nodular-sclerosis type of Hodgkin's lymphoma (Figure 1D). The 9p24.1 amplicon also includes JAK2. Dose-dependent JAK-STAT activity further induces PD-1 ligand transcription. These copy number-dependent mechanisms and less frequently chromosomal rearrangements lead to overexpression of the PD-1 ligands on Reed-Sternberg cells in patients with Hodgkin's lymphoma (66).

The complementary mechanisms of PD-1 ligand overexpression in Hodgkin's lymphoma suggest that this disease may have genetically determined vulnerability to PD-1 blockade. Co-amplification of PDL1 and PDL2 on chromosome 9p24.1 suggests receptor rather than selective ligand blockade as a treatment strategy. The immune escape of malignant cells by upregulation of PDL1 and PDL2 can be at least partially abrogated with checkpoint immunotherapy. With the introduction of antibodies targeting the PD1 receptor on T-cells, the therapeutic outcome for Hodgkin lymphoma patients who relapsed after initial therapy has improved (Figure 1D) (66, 67).

## Novel Approaches for Hematological Malignancies and Future Perspectives

In addition to the standard therapeutic opportunities in hematological malignancies described above which are mainly targeting fusion proteins resulting from genetic translocations, novel drugs targeting the epigenome such as super enhancer blockers including BET and CDK7 inhibitors have recently been discovered and are reviewed elsewhere (19, 68). As the acetylation or methylation pattern is altered in many cancers (69), the U.S. Food and Drug Administration (FDA) has also approved histone deacetylase inhibitors (HDACi) such as vorinostat and DNA methyltransferase inhibitors (DNMTi) such as 5-Azacytidine to treat a small number of hematological diseases (70).

Despite intensive research into the identification of specific SVs apart from translocations as well as into the understanding of the 3D genome structure of malignant cells, these findings could so far not be translated into the clinic and will be subject to further translational research. The application of curaxins, an anti-cancer agent that intercalates into DNA without inducing DNA damage, prevents looping of normally interacting DNA fragments due to the inability of CTCF to bind to its cognate binding sites which in turn affects the spatial genome structure and thus gene expression (71). This promising study interrogating the disruption of long-distance interactions between enhancers and promoters might lead the way to new cancer treatment options targeting the 3D genome organization. Furthermore, it has been shown that a fusion of two TADs accompanied by an almost complete loss of CTCF binding at the boundary between these two TADs leads to an interaction between the MYC promoter and a distal super-enhancer in primary T-ALL samples as well as T-ALL cell lines (72). The increased looping events can be inhibited by using small molecule inhibitors against oncogenic signaling molecules or epigenetic modifiers (72). Another study reveals that a mutation within the EZH2 gene encoding a histone lysine methyltransferase and present among others in non-Hodgkin lymphomas leads to increased H3K27me3 as well as changes in the TAD structure resulting in intra-TAD gene silencing, which can be restored by pharmacological inhibition of the mutant EZH2 (73).

Major challenges regarding the detection of SVs are the prioritization of diagnostic tools for their identification, especially considering limited resources for applying advanced technologies. Moreover, as the presence of multiple targetable SVs in the same patient might offer various treatment approaches, new questions regarding the sequence of therapies become apparent.

## Author Contributions

JS, JR, CK, and CE have drafted the review article and revisited it critically for important intellectual content and accurate citations. JS and CK have substantially contributed to the conception of the review article.

### Conflict of Interest Statement

The authors declare that the research was conducted in the absence of any commercial or financial relationships that could be construed as a potential conflict of interest.
